# Editorial: Genome-wide identification of functional markers to enhance molecular breeding efforts in agriculturally important, underutilized, and less explored plant species

**DOI:** 10.3389/fgene.2025.1627482

**Published:** 2025-08-19

**Authors:** Rajni Parmar, Sapna Thakur, Ram Kumar Sharma

**Affiliations:** ^1^ College of Agriculture, Tennessee State University, Nashville, TN, United States; ^2^ Biotechnology Department, CSIR-Institute of Himalayan Bioresource Technology (CSIR-IHBT), Palampur, Himachal Pradesh, India

**Keywords:** genetic diversity, GWAS-genome-wide association study, molecular markers, sustainable agriculture, molecular breeding

Agriculture is the primary energy source for humans. However, it is facing two major challenges at present: the growing population and rapid climate change, causing extreme weather events that are expected to persist in the future as well. By the end of this century, the global population is expected to grow to reach 10–12 billion, highlighting the importance of increasing total food production to feed the rapidly growing population. Therefore, it becomes very important to accelerate agricultural research with a goal to acquire a deeper understanding of essential traits for future breeding targets. The advances in genomic technologies in the last two decades have contributed immensely to agricultural research. This issue presents a Research Topic of four original research articles that have utilized genome-wide identification of potential genes and association studies to investigate and address several challenging biological questions.

In the current issue, a study done by Liu et al. was reported, wherein genome-wide association analysis was performed in an economically important crop (rice), targeting traits like panicle length (PL), total grain number per panicle (TGP), filled grain number per panicle (FGP), seed setting rate (SSR), and grain weight per panicle (GWP). Elucidation of the underlying genetic players involved in these traits is important for molecular breeding in rice to improve yield. Panicle traits are quantitative in nature and are substantially affected by the environment; therefore, these traits require more comprehensive and extensive research to precisely determine their corresponding Quantitative Trait Loci (QTLs) and underlying genes responsible for phenotypic heterogeneity. The authors performed GWAS on data collected over 3 years and were able to identify four QTLs for PL, three QTLs for FGP, and one QTL for TGP, GWP, and SSR respectively. These QTLs were detected in both GLM (General Linear Model) and MLM (Mixed Linear Model) analysis, and BLUP (Best Linear Unbiased Prediction) was used to improve the accuracy of GWAS via modeling complex interactions between environment and genotypes. Furthermore, two candidate genes, viz. *LOC_Os01g43700* (Cytochrome P450 protein) and *LOC_Os09g25784* (Auxin-induced protein, 5NG4), were also identified as closely associated with PL, and another, *LOC_Os04g47890* (MYB family transcription factor), with FGP, GWP, and TGP. In this study, along with mining of excellent alleles related to panicle traits in rice, 10 parental lines with favorable alleles associated with yield traits were also recommended for future molecular breeding.

Two additional noteworthy studies (Shen et al.; Li et al.) reported in the current issue were on the subjects of wild potato (*S. pinnatisectum*) and common potato (*Solanum tuberosum* L.). Undertaking studies in both wild and cultivated species helps to better understand genetic diversity and to identify beneficial traits which can help in enhancing adaptability and resilience of the crop. This issue includes a study reporting the first draft chromosome-level genome assembly of *S. pinnatisectum*, which has strong resistance against *Phytophthora infestans* and creates genomic resources fundamental for understanding disease resistance mechanisms. A high-quality genome assembly of 664 Mb size with 34,245 genes was reported, and a total of 303 NBS-coding disease resistance genes were also identified. Additionally, it was found that *S*. *pinnatisectum* harbors a high number of unique genes, and its comparative genomic analysis with cultivated species reveals that pathways related to plant-pathogen interaction and phagosomes were highly enriched in wild species, suggesting their putative role in plant resistance. An additional 68 genes involved in resistance to late blight in potato were also reported on the basis of RNA-seq analysis. This study reported a high-quality reference genome assembly using Oxford Nanopore long read sequencing and Hi-C technologies, which has resulted in the creation of a valuable genomic resource of disease resistance genes that can be incorporated in modern breeding programs.

Another important investigation on cultivated potato (*S. tuberosum* L.) in this issue has presented the genome-wide identification and structural characterization of the FBA (Fructose-1, 6-bisphosphate aldolase) gene family (Li et al.). This gene is involved in photosynthesis, energy metabolism, and comprehensive genomic insight into the *StFBA* gene, which can be utilized to improve the efficiency of photosynthesis, tuber development, and abiotic stress response. Two classes (eight Class I and one class II) of *StFBA* genes were reported on the basis of their structure, function, and phylogenetic relationship. The evolutionary relationship of the FBA gene family with six other species, including *Arabidopsis*, rice, tomato, eggplant, tobacco, and wheat, was reported, finding FBA genes of potato and tomato to be closely related. Furthermore, colinearity analyses between potato, tomato, and Arabidopsis revealed that segmental duplication is responsible for the expansion of the potato FBA gene family. After performing promoter and expression analysis, the authors concluded that *StFBA* genes have *cis*-regulatory elements coupled with light and stress response; and blue light increases *StFBA3, StFBA8*, and *StFBA9* expression in stolon, leaf, and tuber. Collectively, the outcome of this study suggests that these genes can be utilized to improve photosynthesis, induction, and expansion of tubers and the abiotic stress response.

The last contribution is a pivotal study published by Kim et al.: it is on Northern red oak (*Q. rubra*), which is an economically and ecologically important tree in North America and Southeastern Canada. The strong and durable hardwood of this tree is used as floorboard and building materials, and *Q*. *rubra* is a key species for woody plant research, as it is one of the most important red oak lumber species. The genome-wide identification and expression analysis of the *PKF* (Phosphofructokinase) gene, which plays a key role in the glycolytic pathway, was carried out. A total of 14 *QrPFK* genes were identified, and phylogenetic analysis divided them into 2 groups: 11 attributed to PFK and 3 to PFP (pyrophosphate-fructose-6-phosphate phosphotransferase). Furthermore, the expression analysis revealed that all 14 *QrPFK* genes have similar expression in leaves; however, stems and roots exhibit differential expression of these genes. This study has uncovered a critical role of *PFK* gene function in *Q. rubra*, laying a foundation for future investigations.

